# Enhancing H11 Protein-Induced Immune Protection Against *Haemonchus contortus* in Goats: A Nano-Adjuvant Formulation Strategy

**DOI:** 10.3390/biology14050563

**Published:** 2025-05-17

**Authors:** Lisha Ye, Simin Wu, Fuqiang Liu, Juan Zhang, Jie Wan, Chunqun Wang, Hui Liu, Min Hu

**Affiliations:** National Key Laboratory of Agricultural Microbiology, College of Veterinary Medicine, Huazhong Agricultural University, Wuhan 430070, China; yls@webmail.hzau.edu.cn (L.Y.); wsm1126@webmail.hzau.edu.cn (S.W.); lfq0523.hzau.edu.cn@webmail.hzau.edu.cn (F.L.); zhang_juan0@webmail.hzau.edu.cn (J.Z.); wj2002@webmail.hzau.edu.cn (J.W.); wangchunqun@webmail.hzau.edu.cn (C.W.); liuhui45@webmail.hzau.edu.cn (H.L.)

**Keywords:** *Haemonchus contortus*, H11, nano-adjuvant, fecal egg count, worm burden, IgG, cytokine, humoral and cellular immunity

## Abstract

The only vaccine against *Haemonchus contortus* is limited by short-lived antibody persistence and the need for frequent booster immunizations. This study leveraged the advantages of nano-adjuvants in enhancing antigen presentation and immune regulation to evaluate the efficacy of novel adjuvants (IMX, AddaS03) and the conventional QuilA combined with H11 protein. Goats were divided into four groups (IMX + H11, AddaS03 + H11, QuilA + H11, and infected control). They were immunized three times and challenged with 6000 infective third-stage larvae (iL3s) of *H. contortus* on the day of the third immunization, with the experiment lasting for 98 days. The results showed that vaccination with IMX + H11 conferred the strongest protection, demonstrating 88.3% efficacy in fecal egg count (FEC) reduction and 75.8% efficacy against worm burden, followed by QuilA + H11 (85.2% FEC reduction and 68% worm burden reduction) and AddaS03 + H11 (79.4% FEC reduction and 61.3% worm burden reduction). Serum IgG analysis revealed high antibody levels in all immunized groups. Cytokine detection found that IMX + H11 significantly upregulated IL-2 and IFN-γ expression in PBMCs and TNF-α expression in splenocytes, activating Th1-type responses and immune memory. QuilA + H11 showed weaker Th1 activation, and AddaS03 + H11 faced limitations due to insufficient antibody persistence for long-term protection. These findings suggest that IMX can induce highly efficient humoral and cellular immunity, providing a new direction for the optimization of *H. contortus* vaccines and suggesting the importance of nano-adjuvants for precise regulation of immune patterns.

## Simple Summary

Haemonchosis, caused by *Haemonchus contortus*, is a major parasitic disease in ruminants. The heavy reliance on anthelmintics has exacerbated drug resistance, making vaccine development imperative. H11 protein, a dominant antigen, confers partial immune protection to the host, but its formulation with the conventional QuilA adjuvant requires periodic booster immunizations. This study aimed to develop novel nano-adjuvants to enhance protective efficacy and prolong protection duration. Findings indicated that an immunostimulating complex matrix (IMX) combined with H11 protein elicited robust humoral immunity and durable immune memory in the host.

## 1. Introduction

*Haemonchus contortus* (*H. contortus*), a major gastrointestinal parasitic nematode in ruminants such as goats and sheep, causes substantial economic losses worldwide [1,2]. Acute infections often lead to host mortality, while subacute and chronic infections result in anemia, growth retardation, and reduced feed conversion efficiency [3,4]. Current control strategies predominantly rely on chemical anthelmintics, but their frequent use has led to the proliferation of drug-resistant strains [5,6,7,8]. To date, only Barbervax^®^, a vaccine composed of native proteins [9,10], has been approved for use in Australia, England, and South Africa. This vaccine contains H11 protein, which is the most immunoprotective antigen of *H. contortus*, and its efficacy against haemonchosis in goats and sheep has been validated in both laboratory and field trials. However, its application remains limited by the short-lived persistence of protective antibodies, necessitating frequent booster immunizations to sustain protection [11,12].

Modern vaccines are composed of antigens and adjuvants, with adjuvants serving as critical components to enhance the immunogenicity of antigens [13,14,15,16,17]. Vaccine research today not only focuses on the identification of potent antigens but also increasingly emphasizes the exploration of novel, more efficacious adjuvants. Nanomaterials demonstrate significant potential as vaccine adjuvants due to their unique biological properties. Their particle size characteristics are highly similar to those of microorganisms, which enable antigen-presenting cells (APCs) to more effectively phagocytose antigen-carrying nanoparticles through pattern recognition receptors (PRRs), thus significantly enhancing the intracellular delivery efficiency of proteins or polypeptide antigens, thereby amplifying immune responses [18]. On one hand, nanoparticles act as delivery carriers that utilize biomimetic structures to overcome physiological barriers, thereby improving APCs’ phagocytic efficiency of antigens [19,20]. On the other hand, they function as immunomodulators whose surface topological structures and chemical compositions can activate toll-like receptor (TLR) signaling pathways. Through various transduction mechanisms, these nanoparticles stimulate the maturation and differentiation of dendritic cells (DCs), establishing a cascade activation network bridging innate immunity to adaptive immunity [21]. Ultimately, this process facilitates the precise directional differentiation of memory T/B lymphocytes, achieving long-lasting protective immunity against specific pathogens.

Through in vitro experiments with goat PBMCs and in vivo studies in non-host animal models (mice), our recent work demonstrated that the comprehensive efficacy of the nano-adjuvant immunostimulating complex matrix (IMX) and AddaS03 surpassed that of lipid nanoparticles (LNPs) [22]. Consequently, IMX and AddaS03 were selected for the immunoprotection experiment in goats, aiming to optimize the H11 protein-based immunization strategy from an adjuvant perspective.

## 2. Materials and Methods

### 2.1. Experimental Animals

Goats with an average age of 4–6 months were purchased from Hubei Biyi Goat Technology Co., Ltd. (Xiangyang, China). All goats were reared in the Laboratory Animal Center of Huazhong Agricultural University, where they were group-penned according to gender and experimental groupings. Throughout the study period, all animals received *ad libitum* access to fresh water and pasture grass, supplemented with measured quantities of corn pellets and wheat bran to ensure adequate nutritional provision under standardized feeding protocols. Prior to the initiation of animal experiments, all goats underwent standardized deworming procedures involving a single dose of levamisole (7.5 mg/kg; Longyu Biotechnology, Wuhan, China) followed by three consecutive administrations of sulfachloropyrazine sodium (12 mg/kg; Youxin Biotechnology, Binzhou, China). Subsequent parasitological monitoring conducted two weeks post-treatment through triplicate fecal examinations using the saturated salt flotation method consistently demonstrated complete absence of parasitic egg shedding. The experimental protocol was approved by the Animal Research Ethics Committee of Huazhong Agricultural University (permit HZAUGO-2024-0007, dated 20 October 2024).

### 2.2. Parasites

Infective third-stage larvae (iL3s) and adult worms of *H. contortus* (Haecon-5 strain, our laboratory-preserved standard strain) were collected [23]. Briefly, fecal samples from goats 21 days post-infection with *H. contortus* were incubated at 25 °C for 7 d, followed by isolation of iL3s from the fecal cultures. Adult worms were collected from the abomasum of infected goats.

### 2.3. Preparation of Antigens and Nanoparticles

H11 protein was purified from adult *H. contortus* using ConA lectin-agarose affinity chromatography (Vector Laboratories, Newark, CA, USA) [24,25]. In brief, adult worms were homogenized in phosphate-buffered saline (PBS) supplemented with 1.0% (*v*/*v*) Tween 20 for 30 min. The homogenate was subjected to centrifugation at 2500× *g* for 20 min, after which the pellet was re-suspended in PBS containing 1.0% (*v*/*v*) Tween 20 and centrifuged again. The resulting precipitate underwent four sequential extraction cycles with PBS containing 1.0% (*v*/*v*) nonaethylene glycol monododecyl ether (Sigma-Aldrich, St. Louis, MO, USA), with supernatants collected at each step. Supernatants were filtered through a 0.22 μm membrane and subjected to affinity purification via ConA lectin-agarose. Bound proteins were extensively washed with a 0.25% (*v*/*v*) Triton X-100 buffer, followed by elution using a buffer containing 200 mM methyl-α-D-mannopyranoside (Sigma-Aldrich, St. Louis, MO, USA) and 200 mM methyl-α-D-glucopyranoside (Sigma-Aldrich, St. Louis, MO, USA). The harvested eluent was dialyzed against PBS for 48 h and finally concentrated with sucrose. Protein concentration was quantified using a BCA assay kit (Beyotime, Shanghai, China).

IMX was prepared through dialysis of QuilA (InvivoGen, San Diego, CA, USA) combined with cholesterol (Sigma-Aldrich, St. Louis, MO, USA) and L-α-phosphatidylcholine (Sigma-Aldrich, St. Louis, MO, USA) [26,27]. Specifically, 5 mg of L-α-phosphatidylcholine and 5 mg of cholesterol were dissolved in 0.5 mL of 20% N-decanoyl-N-methylglucamine (Mega-10) (Aladdin, Shanghai, China) under gentle heating to form a lipid solution. Concurrently, 25 mg of QuilA was dissolved in 1.25 mL of PBS (pH 6.2). The lipid solution (0.5 mL) was subsequently diluted with 3.25 mL PBS and mixed with the QuilA solution. After ice-bath ultrasonication, the mixture was incubated at room temperature for 2 h. Following 60 h PBS dialysis, the product was filtered through a sterile 0.22 μm membrane. The final IMX concentration was standardized to 5 mg·mL^−1^ based on QuilA content. Particle size and zeta potential were characterized by use of a Malvern Zetasizer (Malvern Panalytical, Almelo, The Netherlands). The AddaS03 nano-adjuvant was purchased from Invivogen (InvivoGen, San Diego, CA, USA).

### 2.4. Vaccination Trials

**Figure 1:**
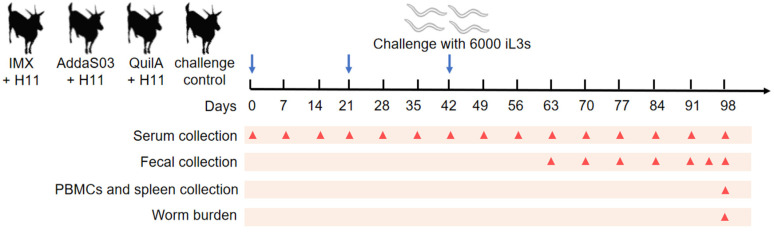
Animal experiment procedure. Four groups of six goats each were vaccinated with the following: IMX + H11, AddaS03 + H11, QuilA + H11, and PBS alone (control). 1. All goats were subcutaneously injected three times (days 0, 21, and 42; blue arrows) at three-week intervals and challenged with 6000 iL3s on day 42. The time points for serum collection (15 times), fecal collection (7 times), PBMCs, and spleen collection, as well as worm collection (once), are indicated by red triangles.

The goats were grouped according to gender and body weight, ensuring three male goats and three female goats in each group, with average body weights maintained at approximately 19 kg for males and approximately 22 kg for females across four designated groups: the IMX + H11 group received immunization with 5 μg/dose of H11 protein mixed with IMX; the AddaS03 + H11 group was immunized with 5 μg/dose of H11 protein combined with AddaS03; the QuilA + H11 group received 5 μg/dose of H11 protein mixed with QuilA; and the infected control group was immunized with PBS alone. As outlined in Figure 1, all animals were subcutaneously vaccinated three times at 3-week intervals (days 0, 21, and 42). On the day of the third vaccination (day 42), all goats were orally infected with 6000 *H. contortus* iL3s using a stomach tube. One goat in the AddaS03 + H11 group died on day 95 due to rumen obstruction caused by accidental plastic ingestion.

### 2.5. Parasitology

From day 63 of the trial (21 days post-infection) until the endpoint (day 98), fecal egg counts (FEC) were monitored regularly. Eggs per gram (EPG) of feces were quantified using the McMaster counting method [28]. Briefly, 2 g of fecal sample was homogenized in 58 mL of saturated sodium chloride solution. The mixture was filtered through a 100-mesh sieve, and the filtrate was thoroughly mixed and aspirated into both chambers of the McMaster counting slide. After settling for 5 min, eggs in both chambers were counted under a microscope. The total egg count was multiplied by 100 to determine the EPG. Each sample was analyzed in triplicate. Finally, the mean cumulative FEC was calculated for each group and the egg reduction rate (%) was analyzed by a calculation [(the mean FEC value for control − mean FEC value for test group)/mean FEC value for control × 100%]. On day 98, all goats were euthanized by intravenous injection of pentobarbital sodium (100 mg/kg). After confirming death through standard procedures, complete abomasa were collected during necropsy. Adult worms were carefully isolated from both the abomasal mucosa and luminal contents before final counting. The worm burden reduction (%) was calculated using the formula [(the mean worm burden for control − the mean worm burden for test group)/mean worm burden for control × 100%].

### 2.6. Detection of Serum IgG Antibody

During the experimental period, serum samples were collected weekly through jugular venipuncture using sterile vacuum tubes (*n* = 15). After allowing blood samples to clot at room temperature for precisely 30 min, serum was separated by centrifugation at 2000× *g* for 10 min at an ambient temperature before being aliquoted into cryovials for preservation. Serum IgG antibody responses to Con A-purified proteins were assessed by enzyme-linked immunosorbent assay (ELISA) following the published protocol [29]. In brief, proteins (3 μg/mL in 50 mM carbonate buffer, pH 9.6) were coated on microtiter plates (Thermo Fisher Scientific, Waltham, MA, USA) and incubated overnight at 4 °C. Plates were washed with PBST (1×), blocked with 1% BSA in PBS for 2 h at 37 °C, and then incubated with diluted serum (1:2000 in PBS, 100 μL/well) for 1 h at 37 °C. Subsequently, horseradish peroxidase (HRP)-conjugated anti-goat IgG secondary antibodies (1:500 dilution) (Beyotime, Shanghai, China) were added and incubated for 40 min at 37 °C. Finally, tetramethylbenzidine (TMB) substrate was added for color development, and the optical density (OD) values were measured at 450 nm.

### 2.7. Isolation of Goat PBMCs and Spleen Cells

Systemic immune responses were assessed using peripheral blood mononuclear cells (PBMCs). At the experimental endpoint (day 98), PBMCs were isolated from goat jugular venous blood collected in sterile EDTA-anticoagulated tubes (5 mL per sample) using a PBMC isolation kit (Haoyang Biotech, Tianjin, China). To evaluate tissue-specific immunity, spleens were collected immediately after euthanasia. Each spleen was dissected into small fragments, snap-frozen in liquid nitrogen, and homogenized using an electric tissue grinder (Beyotime, Shanghai, China) prior to RNA extraction.

### 2.8. Detection of the Transcription Levels of Cytokine-Encoding Genes

Total RNA was extracted from goat PBMCs and splenocytes using an RNA isolation kit (Beyotime Biotechnology, Shanghai, China), followed by cDNA synthesis with a reverse transcription kit (Tiangen Biotech, Beijing, China) according to manufacturers’ protocols. The expression levels of the target genes were quantified using the CFX384 Touch Real-Time PCR Detection System (Bio-Rad, Hercules, CA, USA) following the manufacturer’s protocol. Specific primers for the endogenous reference gene GAPDH and goat target genes are listed in Table 1. Relative gene expression levels were calculated using the 2^(−ΔΔCt)^ method, with the PBS control group normalized to a value of 1.

### 2.9. Statistical Analysis

Statistical analyses were performed using GraphPad Prism 8.0 software (GraphPad Software, San Diego, CA, USA). Normality was verified using a Shapiro–Wilk test prior to parametric analyses. Intergroup comparisons were assessed by one-way ANOVA to determine significant differences. Significance levels are indicated by asterisks in the figures: * *p* < 0.05, ** *p* < 0.01, *** *p* < 0.001, **** *p* < 0.0001.

## 3. Results

### 3.1. Fecal Egg Count (FEC)

**Figure 2:**
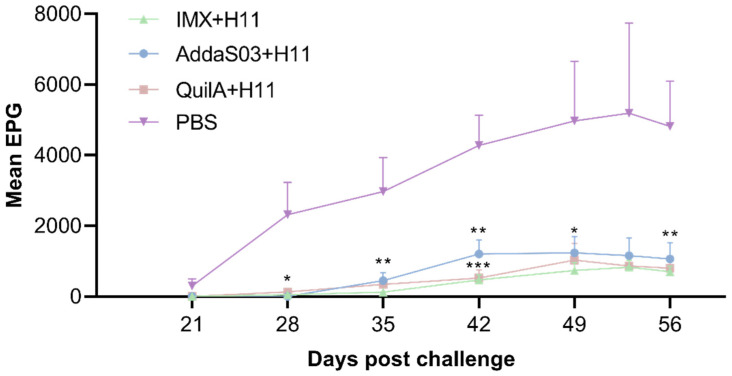
Dynamic change of fecal eggs per gram (EPG) in goats after challenge. Mean EPG (mean ± SD; PBS control: n = 6; IMX + H11: n = 6; AddaS03 + H11: n = 5; QuilA + H11: n = 6) was assessed at seven time points (days 21–56 post-challenge). Statistical significance was determined by one-way ANOVA (* *p* < 0.05, ** *p* < 0.01, *** *p* < 0.001).

During the entire infection period, we observed that all three vaccinated groups exhibited significantly lower fecal EPG compared to the infected control group, with no significant differences observed among the vaccinated groups (Figure 2). Notably, *H. contortus* eggs were first detected in goats from the control group at 21 days post-infection, and egg output progressively increased thereafter. In contrast, no eggs were observed in any vaccinated groups on day 21. Limited egg shedding was detected in a small subset of vaccinated animals on day 28, though all vaccinated subjects maintained consistently low EPG levels throughout the experimental period. Compared with the control group, the IMX + H11 group had the highest egg reduction rate of 88.3%, followed by the QuilA + H11 group with 85.2%, while the AddaS03 + H11 group had a relatively lower egg reduction rate of 79.4% (Table 2).

### 3.2. Worm Burdens

**Figure 3:**
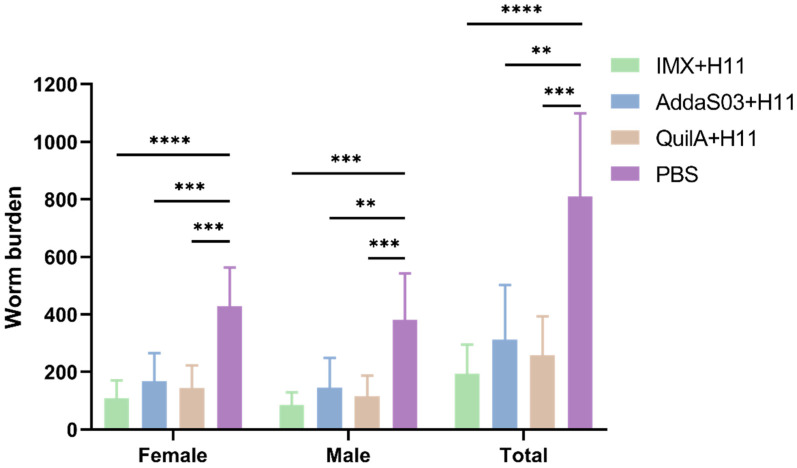
Dynamic range of worm numbers in the trials. Mean group female, male, and total worm numbers (mean ± SD; PBS control: n = 6; IMX + H11: n = 6; AddaS03 + H11: n = 5; QuilA + H11: n = 6) was counted at the end of the trial (day 98). Statistical significance was determined by one-way ANOVA (** *p* < 0.01, *** *p* < 0.001, **** *p* < 0.0001).

At the end of the experiment, abomasal worm burdens were quantified through systematic necropsy following euthanasia. Comparative analysis revealed significant reductions in worm burdens across all vaccinated groups relative to the control group (Figure 3). The IMX + H11, AddaS03 + H11, and QuilA + H11 groups achieved total worm burden reductions of 75.8%, 61.3%, and 68.0%, with no statistically significant differences observed between immunized groups. Specifically, female worm reductions reached 74.5% (IMX + H11), 60.9% (AddaS03 + H11), and 66.5% (QuilA + H11), while male worm reductions were 77.4%, 61.8%, and 69.8% in the respective groups (Table 3).

### 3.3. Serum IgG Antibody Level

**Figure 4:**
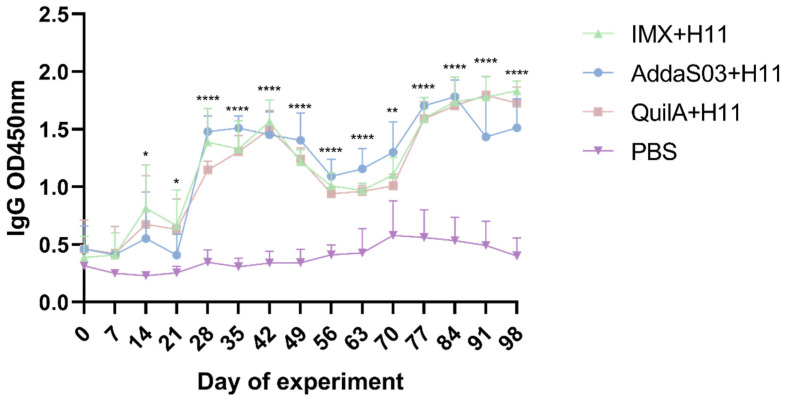
Serum IgG antibody responses in goats vaccinated with PBS alone (control), IMX + H11, AddaS03 + H11, or QuilA + H11 were quantified by enzyme-linked immunosorbent assay (ELISA). Each data point (OD_450_) represents the mean antibody titer (mean ± SD; PBS control: n = 6; IMX + H11: n = 6; AddaS03 + H11: n = 5; QuilA + H11: n = 6). Statistical significance was analyzed by one-way ANOVA (* *p* < 0.05, ** *p* < 0.01, **** *p* < 0.0001).

According to the findings of our prior animal studies, serum IgA levels remained consistently low throughout the low-dose H11 protein immunization protocol, while IgM titers only increased briefly between the second and third immunization doses [33]. These results suggest that neither immunoglobulin class correlated with host protection against subsequent *H. contortus* challenges. Consequently, this study focused on quantifying serum IgG dynamics. Our data demonstrated that H11 protein combined with different adjuvants elicited robust IgG responses, with similar serological profiles observed across all three vaccinated groups (Figure 4). On days 28 to 77 post-immunization, the IgG antibody levels in the AddaS03 + H11 group were higher than those in the other two vaccinated groups, followed by a gradual decline from days 84 to 98. In contrast, the IMX + H11 group achieved the highest endpoint titers among all groups.

### 3.4. Transcription Level Changes of Cytokine-Encoding Genes

Following completion of the animal experiments, cytokine detection was performed using quantitative real-time PCR (qPCR). This analysis was conducted approximately two months after the final immunization, a time frame corresponding to the immune memory phase when antigen-specific T and B lymphocyte memory populations were fully established [34,35]. PBMCs were used to evaluate systemic immune responses, while splenocytes were analyzed to assess localized cellular immunity and T-cell interactions.

**Figure 5:**
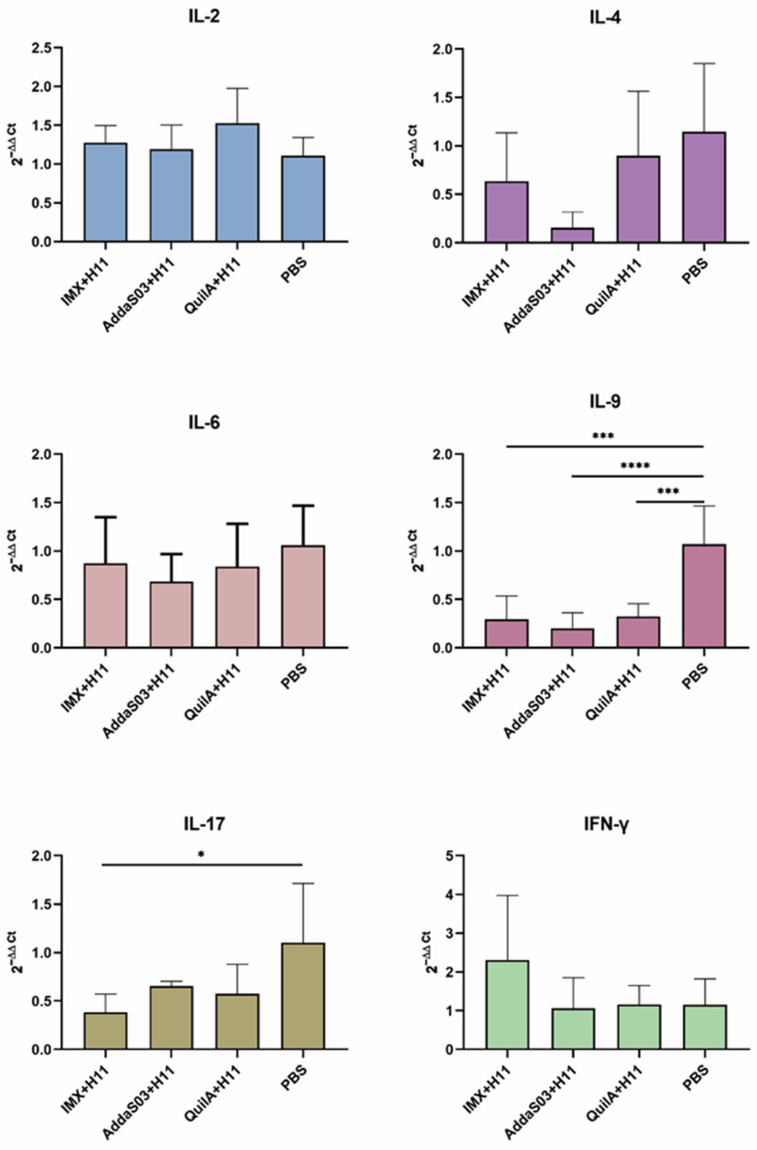
Effects of immunization on cytokine mRNA levels in goat peripheral blood mononuclear cells (PBMCs). Goat PBMCs were collected at the end of the experiment, followed by RNA extraction. Transcript levels of cytokines (IL-2, IL-4, IL-6, IL-9, IL-17, and IFN-γ) were quantified via quantitative real-time PCR. Statistical significance was analyzed by one-way ANOVA (* *p* < 0.05, *** *p* < 0.001, **** *p* < 0.0001).

Analysis of PBMCs revealed that all immunized groups showed a non-significant downregulation trend in IL-4 and IL-6 (*p* > 0.05) and a significant decrease in IL-9 (*p* < 0.001) compared to the infected control group, while only the IMX + H11 group exhibited a significant reduction in IL-17 transcription (*p* < 0.05). In contrast, a moderate upregulation in IL-2 expression was observed in the three immunized groups (*p* > 0.05). Notably, although IFN-γ expression in the IMX + H11 group did not reach statistical significance compared to other antigen-adjuvant combinations (*p* < 0.05), its upward trend may still suggest a potential enhancement of host cellular immune memory (Figure 5).

**Figure 6:**
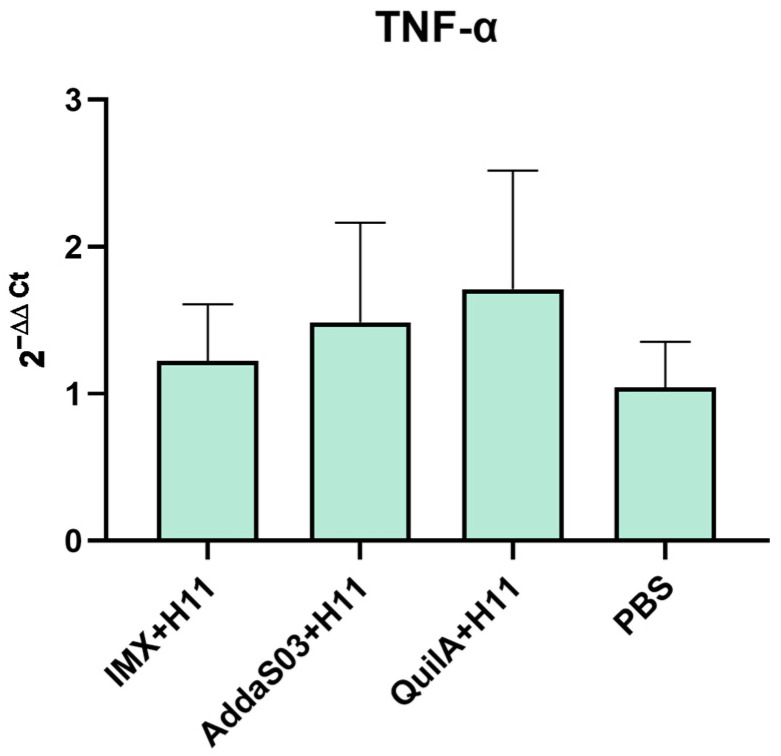
Effects of immunization on cytokine mRNA levels in goat spleen cells. Spleens were collected at the end of the experiment, followed by RNA extraction. Cytokine transcript levels were quantified via quantitative real-time PCR. Statistical significance was analyzed by one-way ANOVA.

Cytokine levels in splenocytes were analyzed. Results revealed that TNF-α mRNA levels were moderately upregulated in all three immunized groups compared to the control group (*p* > 0.05) (Figure 6). These findings suggested that immunization induced the activation of splenic APCs, thereby promoting Th1 cell differentiation and macrophage activation.

## 4. Discussion

In this study, we explored the application of novel nano-adjuvants for vaccines targeting *H. contortus*. Based on preliminary screening results from previous studies [22], two nano-adjuvants (IMX and AddaS03) were selected for evaluation, with the QuilA adjuvant serving as the reference. The results demonstrated that the IMX + H11 formulation achieved relatively higher egg reduction rates and worm reduction rates compared to other formulations. The superior protection conferred by this formulation may stem from the combined effects of humoral and cellular immune responses.

Egg and worm reduction rates serve as critical efficacy indicators for *H. contortus* vaccines [36,37]. In this experiment, the IMX + H11 group demonstrated the highest egg reduction rate (88.3%), followed by the QuilA + H11 group (85.2%) and the AddaS03 + H11 group (79.4%). At trial termination, the corresponding worm reduction rates were recorded as 75.8%, 68.0%, and 61.3% for the IMX + H11, QuilA + H11, and AddaS03 + H11 groups, respectively. All adjuvants combined with H11 significantly reduced FEC and abomasal worm burdens, aligning with previous findings [38]. *H. contortus* typically initiates egg production 21 days post-infection [39]. Our results revealed delayed parasite development in the immunized goats. Eggs were detected in only a subset of vaccinated animals on day 28, suggesting vaccine-induced inhibition of female worm maturation and oviposition capacity (Figure 2). No statistically significant differences were observed among the immunization groups. Notably, all adjuvants in combination with native proteins exhibited strong immunogenicity, conferring protective efficacy throughout the experimental period.

Serum IgG antibodies were continuously monitored in goats throughout the experimental period. The results revealed a declining trend in antibody titers during the later phases (from days 84 to 98) in the AddaS03 + H11 group, whereas both the IMX + H11 and QuilA + H11 groups maintained upward trajectories. Given the established association between H11 protein-induced immunoprotection and IgG antibody levels [40], parasitological parameters across all immunized groups showed positive correlations with antigen-specific IgG production. The observed reduction in IgG levels in the AddaS03 + H11 group may lead to decreased protective efficacy.

To investigate vaccine-induced cellular immune responses, this study also examined cytokine expression profiles. IL-6, predominantly associated with acute inflammation and chronic inflammatory persistence [41,42,43], showed a downward trend in all immunized groups compared to the infected control group at two months post-immunization, although this difference did not reach statistical significance (*p* > 0.05). The observed pattern may suggest a potential modulatory effect of vaccination on *H. contortus*-induced inflammation, but further studies with larger sample sizes are needed to confirm this biological relevance. Previous studies have documented that *H. contortus* infection triggers Th2-polarized immunity, accompanied by sequential activation of Th9/Th17 responses [32,44,45]. In the later experimental phase, decreased expression of Th2/Th9/Th17-associated cytokines coupled with elevated IL-2 levels (a Th1-related cytokine) was observed in all immunized groups, indicating vaccine-primed Th1-biased cellular immunity. This contrasts with the canonical Th2-dominated anti-parasitic response, and we hypothesize that the observed Th1 polarization may confer compensatory immunity while mitigating excessive inflammation-mediated pathology. Specifically, upregulated IL-2 and IFN-γ expression was detected in PBMCs of the IMX + H11 group during late-stage immunization. IFN-γ serves as a hallmark of antigen-specific CD8+ cytotoxic T lymphocyte (CTL) activation [46,47], while IL-2 is critical for maintaining memory T-cell populations [48,49], collectively suggesting the initiation of cellular immunity and establishment of immunological memory. Splenic macrophages, dendritic cells, and activated T cells are known sources of TNF-α [50,51,52]. Notably, higher TNF-α expression was observed in splenocytes of immunized groups versus infected controls (*p* > 0.05). The detected low-level TNF-α at endpoint without ex vivo stimulation could reflect partial quiescence of residual antigen-presenting cells or localized subclinical inflammatory responses. While this aligns with our hypothesis of vaccine-mediated immune activation, its protective implications require further validation through additional endpoints. Methodologically, these interpretations should be weighed against technical considerations. Cytokine transcription levels were assessed using qPCR. While mRNA quantification reflects transcriptional activity of cytokines, its correlation with protein expression may be confounded by post-transcriptional regulatory mechanisms [53]. Furthermore, the absence of in vitro stimulation during cytokine detection introduced limitations to our findings [54,55].

Current research on *H. contortus* vaccines primarily focuses on recombinant subunit vaccine development. Our research team has attempted multiple expression systems to generate recombinant proteins, which successfully induced elevated antigen-specific IgG antibody titers in hosts but failed to confer effective protection [56]. Therefore, future studies should prioritize overcoming the critical challenge of precisely modulating vaccine-induced immune response profiles. Concurrently, it is imperative to validate through animal experiments whether antibodies elicited by nanoparticle-recombinant protein combinations exhibit immunoprotective efficacy. Furthermore, longitudinal monitoring of CD4+ T-cell subset differentiation and dynamic mucosal IgA variations in goat models is warranted to elucidate whether nanoformulations can impede larval migration via antibody-dependent cellular phagocytosis (ADCP). These investigations aim to establish a novel paradigm for transitioning anti-helminth vaccine evaluation from immunogenicity metrics to practical protection outcomes.

## 5. Conclusions

In this study, immune efficacy was evaluated through integrated parasitological and molecular biological indicators. Our results showed that IMX combined with antigen demonstrated optimal protective efficacy, achieving 75.8% worm burden reduction and 85.2% egg reduction rate. This combinatorial strategy effectively elicited robust humoral immunity alongside moderate Th1-biased cellular immune responses.

## Figures and Tables

**Table 1:** Primers used in quantitative real-time PCR.

Name of Cytokine (Reference No.)	Forward Primer Sequence	Reverse Primer Sequence
GAPDH [30]	CCTGGAGAAACCTGCCAAGT	GCCAAATTCATTGTCGTACCA
IL-2 [31]	CAAACGGTGCACCTACTTCA	AGCTTGAGGTTCTCGGGATT
IL-4 [31]	GTACCAGCCACTTCGTCCAT	GCTGCTGAGATTCCTGTCAA
IL-6 [30]	CGTCGACAAAATCTCTGCAA	TTCCCTCAAACTCGTTCTGG
IL-9 [32]	GATGCGGCTGATTGTTT	CTCGTGCTCACTGTGGAGT
IL-17 [31]	TTGTAAAGGCAGGGGTCATC	GGTGGAGCGCTTGTGATAAT
TNF-α [30]	CAGGGCTCCAGAAGTTGCT	GGGCTACCGGCTTGTTATTT
IFN-γ [30]	TAAGGGTGGGCCTCTTTTCT	CATCCACCGGAATTTGAATC

**Table 2:** Fecal egg counts (FEC) of challenged goats throughout the trial. Fecal samples were collected from goats at seven time points (Figure 1), with *Haemonchus contortus* eggs per gram (EPG) quantified microscopically. Group-specific cumulative FEC (mean ± SD) were calculated to assess infection dynamics.

Groups	Mean FEC	SD	Reduction (%) ^a^
IMX + H11	2911.3	1551.6	88.3 **
AddaS03 + H11	5119.8	4264.8	79.4 *
QuilA + H11	3688.3	3330.0	85.2 **
Control	24,846.0	19,946.8	-

^a^ The reduction (%) = [(the mean FEC value for control − mean FEC value for test group)/mean FEC value for control × 100%]. Statistical significance was performed using one-way ANOVA, * *p* < 0.05, ** *p* < 0.01.

**Table 3:** Worm numbers from challenged goats in the trials. At the end of the experiment (day 98), the mean worm numbers (mean ± SD) of individual goats in the abomasa were counted for each group.

Groups	Total			Female			Male		
	Mean	SD	Reduction (%) ^a^	Mean	SD	Reduction (%) ^a^	Mean	SD	Reduction (%) ^a^
IMX + H11	195.7	101.7	75.8 ****	109.5	60.9	74.5 ****	86.2	43.3	77.4 ***
AddaS03 + H11	313.2	189.7	61.3 **	167.6	98.6	60.9 ***	145.6	103.4	61.8 **
QuilA + H11	259	135	68.0 ***	143.7	79	66.5 ***	115.3	71.6	69.8 ***
Control	810.3	288.1	-	428.8	134.2	-	381.5	161	-

^a^ The reduction (%) = [(the mean worm burden for control − the mean worm burden for test group)/mean worm burden for control × 100%]. Statistical significance was determined by one-way ANOVA (** *p* < 0.01, *** *p* < 0.001, **** *p* < 0.0001).

## Data Availability

The data used and/or analyzed during this study are available from the corresponding author on reasonable request.
